# Workplace Aggression and Burnout in Nursing—The Moderating Role of Follow-Up Counseling

**DOI:** 10.3390/ijerph17093152

**Published:** 2020-05-01

**Authors:** Sylvie Vincent-Höper, Maie Stein, Albert Nienhaus, Anja Schablon

**Affiliations:** 1 Department of Work and Organizational Psychology, Universität Hamburg, 20146 Hamburg, Germany; maie.stein@uni-hamburg.de; 2 Department of Occupational Medicine, Hazardous Substances and Public Health, Institution for Statutory Accident Insurance and Prevention in the Health and Welfare Services, 22089 Hamburg, Germany; albert.nienhaus@bgw-online.de; 3 Competence Centre for Epidemiology and Health Services Research for Healthcare Professionals (CVcare), University Medical Centre Hamburg-Eppendorf (UKE), 20246 Hamburg, Germany; a.schablon@uke.de

**Keywords:** nursing, aggression, violence, burnout, emotional exhaustion, depersonalization, personal accomplishment, post-event assistance, post-event counseling

## Abstract

The aim of this study is to obtain a better understanding of the association between the frequency of nurses’ exposure to workplace aggression from patients and their levels of burnout. In particular, we seek to shed light on the role of the availability of follow-up counseling in organizations after critical incidents in mitigating the adverse relationships between physical and verbal aggression and nurses’ burnout. A total of 582 nurses reported how frequently they had experienced physical and verbal aggression from patients in the last 12 months and whether they had the opportunity to receive follow-up counseling in their organization. In addition, nurses rated the extent to which they experienced each of the three dimensions of burnout (i.e., emotional exhaustion, depersonalization, and personal accomplishment). The results showed that both physical and verbal aggression were substantially related to the burnout dimensions. Furthermore, we found that the availability of follow-up counseling in organizations attenuated the relationships between physical aggression and all three burnout dimensions. While we found that the availability of follow-up counseling moderated the relationship between verbal aggression depersonalization, the moderating effects were not significant for emotional exhaustion and personal accomplishment. The findings indicate that the availability of follow-up counseling might help minimize the adverse impact of exposure to aggression from patients on nurses’ mental health.

## 1. Introduction

Workplace aggression is defined as any “behavior by an individual or individuals within or outside an organization that is intended to physically or psychologically harm a worker or workers and occurs in a work-related context” [1] (p. 191). While workplace aggression may take various forms, an important distinction is between physical and non-physical aggression. Physical aggression describes the use of physical force against someone that may lead to physical and psychological harm (e.g., beating, kicking, slapping, pushing, biting, pinching). Verbal aggression comprises all forms of verbal abuse, such as insults, intimidation, swearing, and derogatory language [2]. Although the terms “aggression” and “violence” are often used interchangeably, workplace violence is a specific form of workplace aggression that refers to behaviors that are intended to cause physical harm.

Physical and verbal aggression from patients, their families, and visitors is a pervasive problem in healthcare settings that has become a major public health issue [3,4]. Nursing is the occupation at most risk of experiencing aggression and violence, with nursing staff in psychiatric settings, emergency rooms, geriatric care facilities, and hospitals reporting the highest prevalence rates [5,6,7]. Meta-analytical findings indicate that the prevalence rate of physical aggression is 36% among nurses and that 67% of nurses have experienced verbal assaults [8]. Another meta-analysis showed that 62% of nurses had been exposed to some form of workplace aggression in the past year, with 43% having experienced non-physical aggression and 24% having experienced physical aggression [9]. A study from Germany found that the prevalence of exposure to aggression in the last 12 months was 80% among healthcare workers, of whom 94% had experienced verbal aggression and 70% had experienced physical aggression [5].

Although the exact percentages differ to some degree across studies, the prevalence rates clearly demonstrate that workplace aggression is a serious issue in healthcare settings. In fact, the seriousness of workplace aggression against nurses is magnified because of the devastating consequences associated with exposure to aggression. Numerous studies indicate that experiences of aggression and violence have severe negative effects on healthcare workers’ mental health [7]. In particular, several studies have shown that nurses’ exposure to workplace aggression and violence is a major risk factor for experiencing burnout [4,9,10,11,12,13,14,15,16,17].

**Figure 1:**
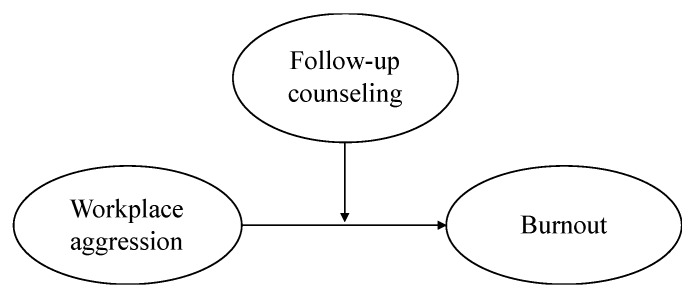
The conceptual model.

In this study, we aim to advance the understanding of the role of the wider organizational context in moderating the association between nurses’ experience of aggression from patients and their levels of burnout. We suggest that the availability of follow-up counseling is an important organizational strategy for minimizing the adverse effects of exposure to workplace aggression on burnout. Figure 1 shows the conceptual model. Specifically, we argue that the relationships between physical and verbal aggression from patients and the three dimensions of burnout (i.e., emotional exhaustion, depersonalization, and personal accomplishment) are weaker when nurses have the opportunity to receive follow-up counseling in their organization after exposure to critical incidents. Considering the role of the organizational context in which aggressive acts occur contributes to obtaining a more complete picture of the associations between nurses’ experiences of physical and verbal aggression from patients and their mental health.

### 1.1. Experiences of Workplace Aggression and Nurses’ Mental Health

Workplace aggression is a source of extreme socio-emotional stress, which may lead to the experience of psychological strain [18]. A large body of research demonstrates that exposure to workplace aggression and violence may severely impair healthcare workers’ mental health, including symptoms of post-traumatic stress disorder, depression, and burnout [7].

As a reflection of psychological strain that is in response to work-related stress, burnout is an important marker of employees’ mental health [19]. According to the most commonly used conceptualization of burnout by Maslach and Jackson [20], burnout comprises the three dimensions of emotional exhaustion, depersonalization, and personal accomplishment. Emotional exhaustion is the energetic component of burnout and refers to feelings of being overextended and depleted of emotional and physical resources. Depersonalization describes the tendency to develop cynical attitudes and feelings about the recipients of one’s care and to respond to them in a detached, callous, and even dehumanized manner. Personal accomplishment is the self-evaluation component of burnout and describes feelings of competence, achievement, and productivity at work. The experience of burnout is associated with reduced feelings of personal accomplishment, which manifests itself in the tendency to evaluate oneself negatively and to be dissatisfied with one’s job accomplishments [19,20].

Several studies have examined the relationships between exposure to workplace aggression and violence and the different dimensions of burnout and found that healthcare workers’ experiences of aggression and violence at work are a major risk factor for burnout [4,9,10,11,12,13,14,15,16,17]. A study of 1163 nurses in Poland showed that exposure to workplace aggression from patients (i.e., physical and verbal assaults, threats, coercion, intimidation, and all forms of harassment) was positively related to emotional exhaustion and depersonalization and negatively related to personal accomplishment. In another cross-sectional study of 1502 nurses in Chinese hospitals, physical and verbal violence at work were shown to be associated with higher levels of emotional exhaustion [9]. A study of 39,894 nurses from 10 European countries showed that high (i.e., weekly to daily) and medium (i.e., monthly) frequencies of violence from patients or relatives were associated with higher levels of burnout [13]. Therefore, we hypothesize the following:

**Hypothesis 1** **(H1).** Physical and verbal aggression are positively related to (a) emotional exhaustion and (b) depersonalization and negatively related to (c) personal accomplishment.

### 1.2. Management of Workplace Aggression and Violence in Nursing

Given the frequency of aggressive acts toward nurses and their severe negative effects on nurses’ mental health, it is critical to understand how organizations may effectively manage incidents of workplace aggression and violence. Although primary prevention should always be given priority, organizations will not be able to prevent all incidents of aggression and violence against nurses. Therefore, it is just as important that organizations know how to prevent or reduce the stress and strain reactions of nurses after an assault occurs.

Post-event interventions (e.g., debriefing, counseling) are widely considered an effective strategy for minimizing potentially negative psychological consequences of exposure to aggression and violence [21,22,23]. While post-event interventions may take various forms, the general idea of such offers is to assist employees in coping with the stress arising from experiences of workplace aggression through the provision of social support [24]. However, empirical evidence on the effectiveness of post-event interventions for managing aggression and violence in terms of healthcare workers’ mental health is very limited [21,22]. In one of the few studies on post-event interventions in healthcare settings, informal counseling and debriefing sessions after experiences of violence were found to improve healthcare workers’ reporting of violent incidents, awareness of risks for violence, avoidance of violent situations, and violence management skills [25]. However, this study did not examine whether the intervention was beneficial in terms of reducing the negative consequences for healthcare workers’ mental health. In their study of 225 employees in a healthcare setting, Schat and Kelloway [24] found that post-event support received from coworkers, supervisors, and management buffered the relationships between different forms of aggression (i.e., physical violence, vicariously experienced violence, and psychological aggression) and emotional well-being.

Therefore, we argue that the availability of follow-up counseling in organizations may help mitigate the negative impact of exposure to workplace aggression from patients on nurses’ mental health. Specifically, we propose that this form of aggression management, which is integrated into organizational structures and processes, may moderate the adverse relationships between nurses’ experiences of physical and verbal aggression and their levels of burnout such that the relationships are weaker when organizations offer follow-up counseling after critical incidents. Therefore, we hypothesize the following:

**Hypothesis 2** **(H2).** The relationship between aggression and burnout is moderated by the availability of follow-up counseling such that follow-up counseling after exposure to critical incidents attenuates the relationships between (a) physical and (b) verbal aggression and all three dimensions of burnout (i.e., emotional exhaustion, depersonalization, and personal accomplishment).

## 2. Materials and Methods

### 2.1. Sample and Procedure

This study received approval from the Hamburg Medical Chamber ethics committee study (reference number: PV5405). Participants were informed that participation was voluntary. To ensure anonymity, we did not ask for names or other personal information. Data were collected using a paper-and-pencil survey. We selected a 10% random sample of hospitals, inpatient and outpatient geriatric care, and facilities for people with disabilities in Germany, which are insured by the Statutory Accident Insurance of the Health and Welfare Service (BGW). In all, 4852 employees received a questionnaire that had to be returned in a self-addressed envelope. In total, 1984 employees working in healthcare and social welfare having direct contact with patients, clients, or residents returned the questionnaire [5]. This large sample comprises a heterogeneous workforce. In our study, we focus on a subsample of nurses (*n* = 582) to gain knowledge on and derive specific implications for this target group.

The majority of nurses were female (80%). A total of 23% were younger than 29 years, 17% were between 30 and 39 years, 26% were between 40 and 49 years, 28% were between 50 and 59 years, and 6% were older than 60 years. Of the participants, 11% worked 20 h or fewer per week, and 18% worked between 21 and 30 h per week. More than half (56%) worked between 31 and 40 h per week, and 15% worked more than 40 h per week. A total of 14% had worked as a nurse for fewer than six years; 21% had worked between 6 and 15 years, and 62% had worked more than 15 years. Nearly one-fifth (17%) of the nurses held a leadership position.

### 2.2. Measures

We assessed workplace aggression with one item each for physical and verbal aggression. The items asked the respondents to indicate how often they had experienced physical and verbal aggression from patients or clients in the last 12 months [5]. The response scale ranged from 1 (“never”) to 6 (“daily”).

Follow-up counseling was assessed with one item asking whether the respondents had been afforded the opportunity to receive counseling in their organization after stressful events [5]. The response scale was dichotomous (0 = no; 1 = yes).

To assess burnout, we used the Maslach Burnout Inventory (MBI) [20]. We assessed emotional exhaustion with 9 items (Cronbach’s α = 0.91, McDonald’s ϖ = 0.91). A sample item for emotional exhaustion is “I feel emotionally drained from my work.” Depersonalization was assessed with 5 items (Cronbach’s α = 0.71, McDonald’s ϖ = 0.72). A sample item for depersonalization is “I worry that this job is hardening me emotionally.” Personal accomplishment was measured with 7 items (Cronbach’s α = 0.79, McDonald’s ϖ = 0.79). A sample item for personal accomplishment is “I have accomplished many worthwhile things in this job.” Responses were scored on a Likert scale ranging from 1 (“never”) to 6 (“very often”).

### 2.3. Statistical Analyses

The data were analyzed using SPSS version 26. To test the hypotheses, we computed stepwise ordinary least squares regression analyses. The study variables were centered at their respective means to enable a meaningful interpretation of the coefficients. In Model 1 of the stepwise regression analyses, we regressed the respective burnout dimension on physical/verbal aggression. In Model 2, we included follow-up counseling as a predictor of burnout. Model 3 additionally included the interaction term, which we built by multiplying physical/verbal aggression and follow-up counseling together. Models 2 and 3 were used to calculate the effect size of the interaction effects ƒ^2^, the ratio of variance uniquely accounted for by the interaction term relative to the unexplained variance in the model including the interaction term [26].

We considered controlling for the effects of several demographic variables (i.e., sex, age, working hours per week) by including them in the regression analyses. To determine whether the results changed when including the control variables, we followed recommendations on the use of control variables [27] and computed all models with and without sex, age, and working hours per week.

## 3. Results

### 3.1. Descriptive Statistics

Table 1 shows the descriptive statistics and intercorrelations of the study variables. Cronbach’s alpha values appear on the diagonal and indicate acceptable internal consistency for all variables. In support of Hypothesis H1, physical and verbal aggression were positively correlated with emotional exhaustion and depersonalization and negatively correlated with personal accomplishment, indicating that nurses who experienced higher frequencies of physical and verbal aggression reported higher levels of burnout.

### 3.2. Regression Analyses

The analyses revealed that the patterns of findings were essentially the same for the models with and without control variables (i.e., sex, age, and working hours per week). Therefore, we report only the results for the models without control variables. Table 2 displays the results of the regression analyses. To test Hypothesis H1, we computed a series of models in which we regressed the respective burnout dimension on physical/verbal aggression (Models 1a–c). The results of these models showed that both forms of aggression were positively related to emotional exhaustion (β = 0.18, *p* < 0.001 for physical aggression and β = 0.21, *p* < 0.001 for verbal aggression) and depersonalization (β = 0.24, *p* < 0.001 for physical aggression and β = 0.24, *p* < 0.001 for verbal aggression), and negatively related to personal accomplishment (β = −0.14, *p* = 0.001 for physical aggression and β = −0.17, *p* < 0.001 for verbal aggression), providing support for Hypothesis H1.

**Figure 2:**
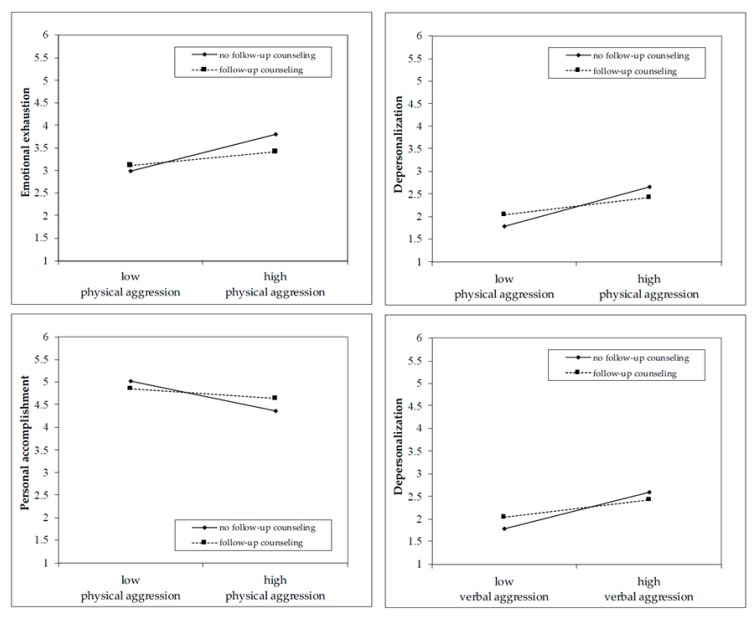
The moderating effects of follow-up counseling.

Moreover, the analyses revealed that follow-up counseling moderated the relationships between physical aggression and the three burnout dimensions (Models 3a–c for physical aggression). The results of Model 3a for physical aggression showed that the availability of follow-up counseling buffered the relationship between physical aggression and emotional exhaustion such that the positive relationship was weaker when organizations offered follow-up counseling (vs. no follow-up counseling; β = −0.13, *p* = 0.015, ƒ^2^ = 0.14). Furthermore, the results of Model 3b for physical aggression showed that the positive relationship between physical aggression and depersonalization was weaker when organizations offered follow-up counseling (vs. no follow-up counseling; β = −0.12, *p* = 0.023, ƒ^2^ = 0.16). Follow-up counseling moderated the negative relationship between physical aggression and personal accomplishment (Model 3c for physical aggression) such that the relationship was weaker when organizations offered follow-up counseling (vs. no follow-up counseling; β = 0.11, *p* = 0.036, ƒ^2^ = 0.08). Model 3b for verbal aggression revealed that follow-up counseling moderated the relationships between verbal aggression and depersonalization such that the positive relationship was weaker when organizations offered follow-up counseling (vs. no follow-up counseling; β = −0.10, *p* = 0.034, ƒ^2^ = 0.13). However, we did not find moderating effects for emotional exhaustion (Model 3a for verbal aggression, β = −0.10, *p* = 0.053) and personal accomplishment (Model 3c for verbal aggression, β = 0.09, *p* = 0.07). Thus, we found partial support for Hypothesis H2. Figure 2 displays the forms of the significant interaction effects.

## 4. Discussion

The prevalence of nurses’ exposure to workplace aggression and violence has received considerable research attention [5,8] and the severe consequences for nurses’ mental health have been well documented [7]. This study examined the relationships between workplace aggression and burnout and demonstrated that the frequency of nurses’ exposure to physical and verbal aggression from patients is substantially related to their feelings of emotional exhaustion, depersonalization, and personal accomplishment. Moreover, we found that the availability of follow-up counseling after exposure to critical incidents moderated the relationships between physical aggression and all three dimensions of burnout such that the relationships were weaker when organizations offered follow-up counseling. In the case of verbal violence, we found a moderating effect of the availability of follow-up counseling in organizations for depersonalization. However, we did not find moderating effects for emotional exhaustion and personal accomplishment.

### 4.1. Implications

These findings are consistent with those of previous empirical studies showing that physical and verbal aggression and violence from patients is significantly related to burnout in the healthcare context [4,9,10,11,12,13,14,15,16]. In particular, we found substantial relationships between verbal aggression and all three dimensions of burnout, suggesting the seriousness of verbal aggression in terms of mental health impairments. While physical acts are the most salient and extreme form of aggression, the findings of this study indicate that verbal aggression should not be overlooked [6]. Therefore, organizations should not only focus on preventing physical forms of workplace aggression but should also acknowledge the adverse impact of verbal aggression on nurses’ mental health and aim to prevent this form of aggression from patients.

Moreover, this study contributes to obtaining a more complete picture of the associations between nurses’ experiences of physical and verbal aggression from patients and their mental health by considering the role of the wider organizational context. Specifically, the results provide support for the notion that nurses should have the opportunity to receive assistance from their organizations in the form of follow-up counseling after exposure to physical and verbal aggression from patients to reduce nurses’ risk of experiencing burnout [22].

While follow-up counseling mitigated the adverse relationships between physical aggression and all three burnout dimensions, the moderating effects for verbal aggression were less consistent. The availability of follow-up counseling moderated the relationship between verbal aggression and depersonalization, but we did not find moderating effects for emotional exhaustion and personal accomplishment. This finding might be because nurses are less likely to consider follow-up counseling after exposure to verbal aggression from patients as being necessary because of the tendency to view verbal aggression as a mild form of aggression [28]. Considering that acts of verbal aggression from patients may have an adverse impact on nurses’ mental health, it is important to recognize the severity of verbal aggression and to reduce the barriers to utilizing organizational assistance after exposure to verbal aggression from patients.

Generally, knowledge of how organizations can reduce the negative impact of exposure to workplace aggression and violence on nurses’ mental health is very limited [21,22]. This study adds to the few studies on post-event intervention strategies in healthcare settings [24,25] and advances the understanding of how organizations may mitigate the damages associated with workplace aggression and violence in nursing. In their study in the healthcare context, Schat and Kelloway [24] found that general patterns of support from within the organization (e.g., “My supervisors provide support when I experience an aggressive or violent situation at work”) buffered the relationship between experiences of workplace aggression and emotional well-being. The findings of our study add to the body of knowledge on the post-event management of aggression and violence by demonstrating that follow-up counseling as a more formalized form of support in organizations may help minimize the potential negative effects of exposure to aggression on nurses’ mental health.

Although this study indicates that follow-up counseling may be an effective secondary intervention, it is important to note that preventing the occurrence of aggression and violence in the first place should be the main goal for organizations. Primary prevention may comprise behavioral trainings that convey communication and de-escalation skills and make nurses feel secure and competent when dealing with critical situations [22,29]. Another important approach for preventing workplace aggression toward nurses is the design of working conditions that reduce the risk of aggressive acts (e.g., adequate space, opportunities to safely exit, adequate lightning, and adequate staffing) [30].

### 4.2. Limitations and Future Directions

Despite its contributions, this study is not without limitations that need to be considered when interpreting the findings. First, we cannot draw conclusions about the direction of the effects. That is, we cannot rule out the possibility that nurses’ levels of burnout influence the frequency of experiences of workplace aggression. Future studies should address this issue by using longitudinal study designs [7].

Furthermore, all study variables were assessed using self-reports, which may raise concerns about common method variance [31]. Although it is possible that common method variance has inflated the correlations of the study variables, it is important to note that common method variance is unlikely to inflate interaction effects [32]. Nonetheless, future research should aim to prevent common method variance by collecting data from different sources (e.g., supervisors and coworkers) and by using different methods (e.g., qualitative assessments).

Verbal and physical aggression were measured with single items that assessed the frequency of nurses’ experiences of verbal or physical aggression in the past 12 months. Thus, we did not collect information on the intensity and quality of the experienced acts of aggression although it is likely that these factors influence the strength of the relationship between experiences of aggression from patients and burnout [10,33]. Furthermore, we could not examine whether the characteristics of the perpetrator (e.g., the patient’s gender) or the specific situation in which the aggressive act occurred (e.g., whether nurses were alone with the patient) affect the relationship between experiences of aggression and burnout. Finally, we did not consider the causes of and motives behind the acts of aggression (e.g., harmful intention, self-blame, reaction to medication, symptom of the patient’s disease). However, research on workplace aggression indicates that the victim’s attributions may moderate the relationships between workplace aggression and strain [33].

To overcome the limitations of the single-item measures, future research would benefit from conducting mixed-methods studies. For example, quantitative surveys that assess the frequency of nurses’ exposure to aggression and violence from patients should be combined with qualitative assessments that explore the specific qualities and circumstances of the aggressive act (e.g., critical incidence technique). Applying mixed-methods studies may contribute to obtaining a more complete picture of the processes and mechanisms underlying the adverse impact of exposure to aggression from patients on nurses’ mental health.

The results give credit to the notion that the availability of follow-up counseling is especially helpful in mitigating the negative effects of physical aggression. However, the substantial association between verbal aggression and nurses’ mental health in this study indicates the importance of developing interventions that counteract the adverse effects of verbal aggression. Qualitative techniques can be useful for identifying the different needs after exposure to physical aggression and after exposure to verbal aggression to make it possible to offer suitable post-event interventions.

Finally, the specific contents of post-event intervention strategies that help mitigate the negative impact of workplace aggression on nurses’ mental health remain unclear. In this study, we assessed the availability of follow-up counseling, which may comprise a variety of different offers of assistance (e.g., feedback, support, debriefing, professional assistance). Moreover, we asked the nurses for the general availability of follow-up counseling in their organizations and not for their actual utilization of follow-up counseling after exposure to critical incidents. It is conceivable that the availability of post-event assistance is an indicator of the overall organizational safety culture and that this culture, rather than the availability of follow-up counseling, is responsible for the moderating effects. Thus, it is necessary to further explicate how and why follow-up counseling moderates the relationship between exposure to workplace aggression and burnout. Therefore, we recommend that future studies specify the contents of follow-up counseling, measure the actual utilization of post-event assistance, and consider the moderating role of organizational safety culture. To give concrete and evidence-based recommendations regarding the management of aggression and violence from patients, longitudinal and evaluation studies that determine the effectiveness of different post-event interventions are needed [21]. Therefore, future studies should conduct high-quality intervention studies (e.g., RCTs) to rigorously evaluate the effectiveness of different offers for assistance after exposure to workplace aggression.

## 5. Conclusions

Workplace aggression from patients is a prevalent issue in nursing that may have severe consequences for nurses’ mental health. The availability of follow-up counseling in organizations seems to be an effective post-event intervention strategy for reducing the adverse effects of nurses’ experiences of aggression from patients on their mental health. Specifically, this study provides support for the notion that receiving assistance from the organization in the form of follow-up counseling after exposure to physical and verbal aggression from patients may reduce nurses’ risk of experiencing burnout. Further research is needed to rigorously evaluate the effectiveness of such secondary intervention strategies. The findings of this study not only have implications for nurses’ mental health but also for organizational outcomes, such as the quality of patient care and intent to leave [13,34], and for the profession’s ability to attract and retain nurses within the healthcare system.

## Figures and Tables

**Table 1:** Descriptive statistics and correlations.

	*M*	*SD*	1	2	3	4	5
1. Physical aggression	2.53	1.51					
2. Verbal aggression	3.51	1.67	0.69 ***				
3. Follow-up counseling ^1^	0.28	0.45	0.20 ***	0.18 ***			
4. Emotional exhaustion	3.26	0.99	0.18 ***	0.21 ***	−0.05		
5. Depersonalization	2.21	0.82	0.24 ***	0.24 ***	0.05	0.50 ***	
6. Personal accomplishment	4.74	0.60	−0.14 **	−0.17 ***	0.00	−0.33 ***	−0.43 ***

Note: *N* = 582; 1 = follow-up counseling; ** *p* < 0.01; *** *p* < 0.001.

**Table 2:** Results of the regression analyses.

	Emotional Exhaustion						Depersonalization						Personal Accomplishment					
	Model 1a		Model 2a		Model 3a		Model 1b		Model 2b		Model 3b		Model 1c		Model 2c		Model 3c	
	β	*SE*	β	*SE*	β	*SE*	β	*SE*	β	*SE*	β	*SE*	β	*SE*	β	*SE*	β	*SE*
**Physical aggression**																		
Intercept	3.26	0.04	3.32	0.05	3.33	0.05	2.21	0.03	2.21	0.04	2.22	0.04	4.74	0.02	4.73	0.03	4.72	0.03
PA	0.18 ***	0.03	0.20 ***	0.03	0.28 ***	0.03	0.24 ***	0.02	0.25 ***	0.02	0.32 ***	0.03	−0.14 **	0.02	−0.15 **	0.02	−0.22 ***	0.02
FC ^1^			−0.09*	0.09	−0.07	0.09			−0.01	0.08	0.01	0.03			0.03	0.06	0.02	0.06
PA × FC					−0.13 *	0.06					−0.12 *	0.03					0.11 *	0.03
*R* ^2^	0.03		0.036		0.044		0.058		0.057		0.063		0.018		0.017		0.023	
∆*R*^2^			0.006		0.008				−0.001		0.006				−0.001		0.006	
**Verbal aggression**																		
Intercept	3.26	0.04	3.31	0.05	3.32	0.05	2.21	0.03	2.21	0.04	2.21	0.04	4.73	0.02	4.82	0.03	4.81	0.03
VA	0.21 ***	0.02	0.23 ***	0.02	0.28 ***	0.02	0.24 ***	0.02	0.24 ***	0.02	0.30 ***	0.02	−0.17 **	0.02	−0.17 ***	0.02	−0.22 ***	0.02
FC ^1^			−0.09*	0.09	−0.08 *	0.09			0.00	0.08	0.02	0.08			0.03	0.06	0.02	0.06
VA × FC					−0.10	0.05					−0.10 *	0.04					0.09	0.03
*R* ^2^	0.044		0.050		0.054		0.057		0.056		0.061		0.026		0.026		0.030	
∆*R*^2^			0.006		0.004				−0.001		0.005				0.000		0.004	

Note: *N* = 582; PA = physical aggression; VA = verbal aggression; FC = follow-up counseling; β = standardized coefficients; SE = Standard Error; ^1^ 0 = no follow-up counseling, 1 = follow-up counseling; * *p* < 0.05; ** *p* < 0.01; *** *p* < 0.001.
